# Skin and soft tissue infections and bacteremia caused by Vibrio cincinnatiensis

**DOI:** 10.1016/j.idcr.2022.e01564

**Published:** 2022-07-08

**Authors:** Kotaro Kunitomo, Naomichi Uemura, Taro Shimizu, Satoshi Hayano, Takahiro Tsuji

**Affiliations:** a Department of General Medicine, Kumamoto Medical Center, 1–5, Ninomaru, Kumamoto, Kumamoto, 860-0008, Japan; b Department of Nephrology, Kumamoto Medical University Hospital, Kumamoto 860-8556, Japan; c Department of Diagnostic and Generalist Medicine, Dokkyo Medical University Hospital, Tochigi 321-0293, Japan; d Department of Internal Medicine, Japanese Red Cross Kumamoto Hospital, Kumamoto, Japan

**Keywords:** *Vibrio cincinnatiensis*, Skin and soft tissue infection, Bacteremia

## Abstract

*Vibrio cincinnatiensis* is a halophilic species found in marine and estuarine environments worldwide. It is a rare pathogen whose impact on humans has remained unclear; only two cases of *V. cincinnatiensis* infection have been reported in humans, so far. A 63-year-old man with a history of myocardial infarction and type 2 diabetes mellitus presented to the emergency department with fever and dyspnea. Physical examination demonstrated notable abdominal distension and bilateral lower leg edema. marked abdominal distension and bilateral lower leg edema. The patient was diagnosed with bacteremia and exacerbated heart failure. Blood and skin cultures revealed the presence of the gram-negative pathogen *V. cincinnatiensis*. Combined antibiotic therapy using intravenous tazobactam /piperacillin resulted in a gradual recovery with no recurrence observed at the 9-month follow-up. To the best of our knowledge, this is the third case of *V. cincinnatiensis* infection reported in humans and the first one to be associated with skin and soft tissue infection. We suggest that although *V. cincinnatiensis* is a rare pathogen, it should be considered as a potential infective agent in the differential diagnosis of immunocompromised patients, regardless of any recent exposure to seawater or marine products.

## Highlights

• We report a *V. cincinnatiensis* infection associated with skin/soft tissue infection.
• A 63-year-old man with previous myocardial infarction and diabetes was treated.
• *V. cincinnatiensis* should be considered in diagnosed immunocompromised patients.
• Infection may develop regardless of recent exposure to seawater or marine products.

## Introduction

*Vibrio* spp. naturally exist in marine, estuarine, and brackish waters, and they are mostly transmitted to humans through water and seafood contamination [1]. *Vibrio cincinnatiensis* is a halophilic species, present in marine and estuarine environments worldwide [2], and even in the mussel, *Mytilus galloprovincialis* [3], [4]. It is a rare pathogen whose impact on humans has not yet been fully elucidated; however, it has been isolated from animals and is identified as a possible zoonotic pathogen [1]. In 1986, Brayton et al. reported the first case of *V. cincinnatiensis* infection in humans in a patient with meningitis [5]. Another study reported its presence in the stool specimens of elderly individuals with weakened immunity and suffering from enteritis [6].

## Case

A 63-year-old man with a history of myocardial infarction and untreated type 2 diabetes mellitus, was admitted to the emergency department with fever, chills, shivering, and dyspnea. He had the body temperature of 40.4 °C, blood pressure of 147/99 mmHg, respiratory rate of 30/min, and pulse of 137/min. Physical examination revealed orthopnea, marked abdominal distention, bilateral leg edema, and stasis dermatitis, as well as intact and purulent drainage of the lower legs. He reported gradual worsening of the abdominal distension and bilateral lower leg edema in the past three months. Incidentally, he had not visited the seaside or eaten any seafood prior to his visit.

The initial laboratory test results indicated a white blood cell count of 2.2 × 10^9^/L; hemoglobin level of 10.2 g/dL; platelet count of 22.1 × 10^4^/μL; and a C-reactive protein level of 1.91 mg/dL.

Computed tomography scanning of the chest and abdomen without using contrast revealed bilateral pleural effusion and marked ascites, but the source of fever could not be determined. On the second day, a gram-negative rod was detected in the blood culture using the BACT/ALERT3D blood culture system (bioMérieux). We established a working diagnosis of bacteremia occurring as a secondary complication of the skin and soft tissue infection in the lower legs and the exacerbation of chronic heart failure.

**Fig. 1:**
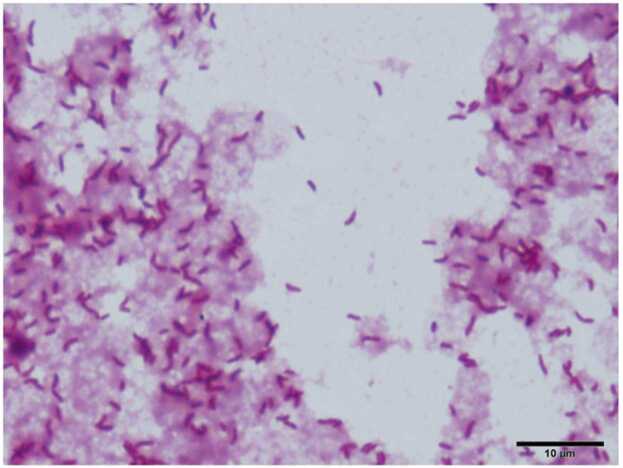
Gram staining of the blood culture indicates the presence of curved gram-negative rods.

Upon admission, empirical therapy with intravenous meropenem (0.5 g; 3 times/day) administration was initiated. However, after detecting the gram-negative rods, antibiotic therapy was changed to intravenous tazobactam/piperacillin (4.5 g; 3 times/day) administration as we suspected *Pseudomonas aeruginosa*-mediated skin infection (Fig. 1).

**Fig. 2:**
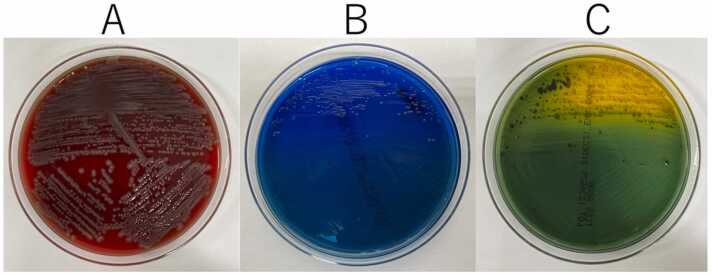
(A) *Vibrio cincinnatiensis* colonies were observed as β-hemolytic and oxidase-positive gram-negative rods on 5 % sheep blood agar after incubation for 20 h at 35 °C. (B) *Vibrio cincinnatiensis* showed poor growth on bromothymol blue (BTB)-lactose agar and (C) small, green colonies appeared on thiosulfate-citrate-bile-salt-sucrose agar after incubation for 16 h at 35 °C.

Two subsequent sets of blood cultures revealed the presence of *V. cincinnatiensis*, while skin cultures were positive for *V. cincinnatiensis* and *P. aeruginosa* (Fig. 2).

The patient completed a 14-day-long intravenous antibiotic course to which both pathogens were determined to be sensitive. He was also administered diuretics for treating the exacerbated chronic heart failure. The clinical symptoms improved upon treatment initiation. On day 22, the patient was discharged from hospital upon completion of the antimicrobial course. No recurrence was observed at the 9-month follow-up.

## Discussion

To the best of our knowledge, this is the third case of *V. cincinnatiensis* infection reported in humans and the first to be associated with skin and soft tissue infection. All three cases have been detected in the elderly, patients with diabetes, or immunocompromised individuals. Although exposure to seawater and seafood is a common risk factor for *Vibrio* spp. infection, for these three *V. cincinnatiensis* infection cases, there was no such history of marine exposure, and the route of infection was unknown.

Here, *V. cincinnatiensis* was detected in blood and skin cultures, indicating that it may have caused the bacteremia. After incubation in 5 % sheep blood agar for 20 h at 35 °C, the isolated microorganisms appeared as β-hemolytic, oxidase-positive, gram-negative rods. The growth was poor on bromothymol blue (BTB)-lactose agar, but small, green colonies were detected on thiosulfate-citrate-bile-salt-sucrose agar after 16 h of incubation at 35 °C. The organism was identified as *V. cincinnatiensis* using matrix-assisted laser desorption/ionization time-of-flight mass spectrometry (MALDI-TOF MS; MALDI Biotyper Version 2.0, Bruker Daltonics, USA) with a score of 2.0. Additionally, a comparison of the 16S rRNA sequences with GenBank sequences confirmed that the isolated strain had 99 % homology with *V. cincinnatiensis* ATCC 35912T (Accession number X74698.1). Antimicrobial susceptibility testing using the broth microdilution method revealed that the microorganism was susceptible to tazobactam/piperacillin, cefotaxime, ceftazidime, cefepime, ciprofloxacin, gentamicin, ampicillin, and cefazolin and resistant to tetracycline and meropenem. Breakpoints for susceptibility testing were determined using the protocol described in the Clinical and Laboratory Standard Institute document M45 third edition [7]. Since *P. aeruginosa* was detected in the skin cultures, there was a possibility of co-existing anaerobic bacteria, hence treatment with tazobactam/piperacillin was continued.

Usually, *Staphylococcus* and *Streptococcus* cause skin and soft tissue infections. For mild infections, penicillin and first-generation cephalosporins are the standard drugs, while in severe cases, broad-spectrum antimicrobials (meropenem + vancomycin or piperacillin/tazobactam + vancomycin) are recommended [6]. Although skin and soft tissue infections caused by gram-negative rods are rare, reports indicate that inappropriate antibiotic therapy, surgical site infection of breast implants, prolonged hospitalization, uncontrolled diabetes mellitus, and freshwater or seawater exposure can pose as potential risk factors [8]. Among the *Vibrio* spp., *Vibrio vulnificus* is the most common cause of skin and soft tissue infection [9], especially in cirrhotic and immunosuppressed patients, while *V. alginolyticus* causes infections in patients with radiodermatitis [10] or at the wound sites [11]. The above-mentioned cases did not present any underlying disease, but had a history of seawater exposure. Interestingly, *V. cincinnatiensis* reportedly causes meningitis and enteritis, but to the best of our knowledge, this is the first reported case of skin and soft tissue infection caused by this pathogen. Although cefazolin is used to treat mild to moderate skin and soft tissue infections, empiric cefazolin treatment may initially lead to treatment failure since *Vibrio* spp. are often resistant to cefazolin. Incidentally, *V. vulnificus* has a better prognosis with early and appropriate treatment with a combination of third-generation cephalosporins and minocycline [6].

In conclusion, *V. cincinnatiensis* is a rare pathogen, and it should be considered a potential infective agent for differential diagnosis in immunocompromised patients, even without any recent exposure to seawater or marine products. Although there are only a few reports of *V cincinnatiensis* infections in humans, we believe that further studies of such reported cases will help to improve the knowledge base related to the pathogenesis of *V. cincinnatiensis*.

## Consent

Written informed consent was obtained from the patient for publication of this case report and accompanying images. A copy of the written consent is available for review by the Editor-in-Chief ofthis journal onrequest.

## Ethical approval

Because this is a case report, ethical approval has not been obtained.

## Financial support

None.

## CRediT authorship contribution statement

Kotaro Kunitomo and Naomichi Uemura identified the significance of this study. Kotaro Kunitomo and Naomichi Uemura wrote the manuscript, Takahiro Tsuji, Taro Shimizu and Satoshi Hayano revised it. All authors approved the final version of the manuscript.

## Conflicts of interest statement

The authors report no conflict of interest.
